# Surgical Management of Oral Squamous Cell Carcinoma With Reconstruction of the Oral Defect Using a Lingual Island Flap: Case Report

**DOI:** 10.1155/crid/9749699

**Published:** 2026-07-30

**Authors:** Julio Humberto Fernandez, Alexis Arcenio Pacha Guerrero, Maria Fernanda Garcia Aguilera, Yunqi Yu

**Affiliations:** ^1^ Department of Surgery, SOLCA Hospital Quito Branch, Quito, Ecuador; ^2^ School of Odontology, Faculty of Health Sciences, International University of Ecuador, Quito, Ecuador; ^3^ Department of Maxillofacial Surgery, School of Odontology, Faculty of Health Sciences, UTE University, Quito, Ecuador, ute.edu.ec; ^4^ Department of Research, SOLCA Hospital Quito Branch, Quito, Ecuador; ^5^ Milken Institute School of Public Health, George Washington University, Washington, DC, USA, gwu.edu

**Keywords:** lingual island flap, oral squamous cell carcinoma, surgical resection

## Abstract

Oral squamous cell carcinoma (OSCC) is the most common malignant neoplasm of the oral cavity and requires surgical resection with adequate oncologic margins and functional reconstruction of the resulting defect. We report the case of a 78‐year‐old male patient with locally recurrent well‐differentiated OSCC who was treated with surgical resection with 10 mm safety margins, reconstruction with an island tongue flap, and selective bilateral Level IA neck dissection. Although microvascular free flaps represent the reconstructive gold standard for extensive defects and the submental flap is a reliable regional alternative, the island tongue flap was selected because of the posterior location of the defect, the moderate size of the resection, and the availability of adjacent tissue with similar characteristics, allowing adequate intraoral coverage with lower surgical morbidity. One of its main disadvantages is the need for a second procedure to divide the pedicle. Postoperative follow‐up demonstrated satisfactory intraoral healing, with no evidence of flap necrosis, wound dehiscence, or scar contracture. Adequate tongue mobility, including protrusion, elevation, and lateralization, was observed, with satisfactory preservation of speech, swallowing, and mouth opening. At the 14 months of follow‐up, the patient remained asymptomatic, with preserved oral function and no clinical evidence of local recurrence. This case demonstrates that the island tongue flap remains an effective and functional reconstructive option for selected posterior intraoral defects, providing favorable medium‐term outcomes, preservation of oral function, and minimal morbidity.

## 1. Introduction

Oral squamous cell carcinoma (OSCC) is the most common malignant neoplasm of the oral cavity, accounting for approximately 90% of tumors in this region. It most frequently affects the tongue, oral mucosa, lips, and jaw [1]. The World Health Organization (WHO) estimates an annual incidence of 657,000 new cases and 330,000 deaths from oral cancer, placing it between the sixth and eighth leading causes of malignant neoplasia worldwide [2].

Its etiology is multifactorial: excessive consumption of tobacco and alcohol, chewing of areca nut, and chronic inflammation of the oral mucosa [3]. Infection by the human papillomavirus (HPV), especially Genotypes 16 and 18, also represents a relevant factor because its oncoproteins E6 and E7 inactivate p53 and pRB, favoring malignant transformation [2]. It predominates in men (2:1) and in developing countries, although in young women who are neither smokers nor drinkers, an increase in cases attributable to HPV is observed [4].

Clinical diagnosis can identify up to 99% of suspicious lesions, but surgical biopsy is the most reliable confirmatory method. Complementary techniques include toluidine blue staining, autofluorescence, and, more recently, salivary biomarkers under investigation [3]. Treatment varies according to stage. Wide surgical resection with oncological margins is the approach of choice, complemented by radiotherapy, chemotherapy, and reconstruction in selected cases [1].

The objective of this work is to present a clinical case of OSCC treated surgically with a reconstructive approach.

## 2. Clinical Case Presentation

### 2.1. Background

A 78‐year‐old male patient with a history of high blood pressure and benign prostatic hyperplasia under treatment was evaluated at the Oncology Surgery Service of Solca Hospital, Quito, in December 2024.

The patient reported the presence of an ulcer in the right retromolar region since August 2022. He was initially evaluated at another health unit, where an incisional biopsy was performed, revealing well‐differentiated squamous cell carcinoma of the right oral vestibule, clinical stage T1N0M0. At that time, it was decided to perform surgical excision of the lesion with selective lymph node dissection, a procedure that was carried out successfully.

### 2.2. Current History

Ten days before the evaluation, the patient reported the appearance of a lesion in the right oral vestibule, for which he attended the Solca Oncology Hospital in Quito. Clinical examination revealed a cervical scar consistent with prior right supraomohyoid dissection. A 1.5 cm, mobile, indurated nodule was palpated at ipsilateral Level IA. A broad‐based, elevated, verrucous lesion, approximately 10 mm in the posterior third, with poorly defined borders and a leukoplakia‐like appearance, was identified in the right oral vestibule.

An incisional biopsy of the lesion confirmed the presence of well‐differentiated, invasive squamous cell carcinoma. Additionally, fine‐needle aspiration (FNAB) of the cervical node revealed positive lymph node metastasis at right Level IA.

**Figure 1:**
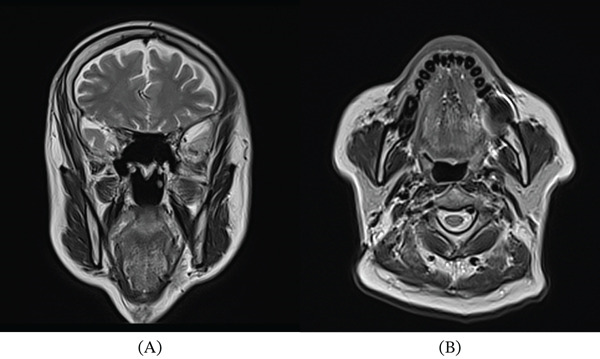
S/c MRI of the T2 face (January 12, 2025). (A) Coronal and (B) axial section.

Facial MRI revealed a nodular lesion with an internal hyperintense component, located in the right anterior and lateral hard palate, extending toward the ipsilateral tonsillar pillar, consistent with local recurrence. The lesion measured 7.2 × 6.5 mm in diameter on the axial axes and 7.2 mm on the craniocaudal axis (Figure 1).

### 2.3. Additional Tests

**Figure 2:**
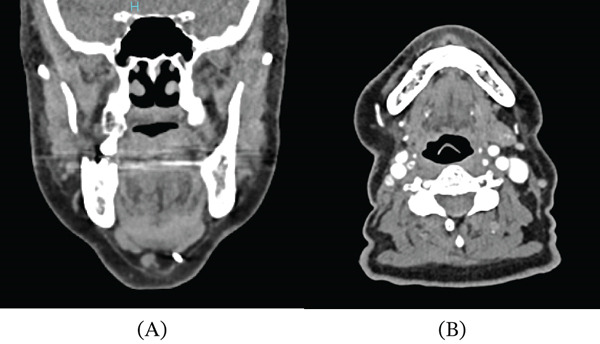
CT scan of the facial mass (January 15, 2025). (A) Coronal section and (B) axial section.

Extension studies were performed such as a simple and contrast‐enhanced computed tomography (Figure 2) and preoperative examinations, the results of which were within normal parameters.

### 2.4. Surgical Intervention

He was evaluated by maxillofacial surgery, with prior informed consent, and the excision of squamous cell carcinoma of the right buccal mucosa was planned, along with an intraoperative study to determine lesion‐free edges, excision of Teeth 18 and 47, reconstruction of the defect with a tongue rotation flap, and selective bilateral Level IA emptying.

The surgical procedure was performed in February 2025 in 2 surgical stages.

#### 2.4.1. First Surgical Time

**Figure 3:**
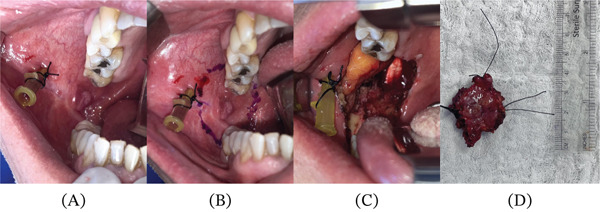
Initial surgical procedure: (A) Stensen′s canal canalization, (B) demarcation of the lesion canalization, (C) oral mucosal defect canalization, and (D) lesion sample for intraoperative.

**Figure 4:**
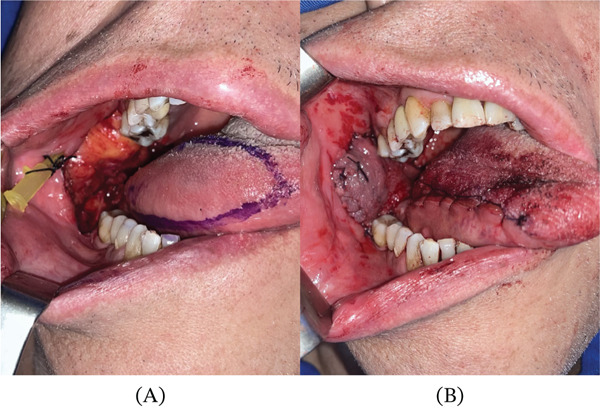
Reconstruction of the oral mucosal defect. (A) Demarcation of the lingual island flap canalization. (B) Layered synthesis preserving the vascular pedicle.

Under general anesthesia, the surgical procedure was initiated with cannulation of the Stensen′s duct and delineation of the lesion with 10 mm safety margins circumferentially around the tumor. Teeth 18 and 47 which were involved within the planned oncologic margins were extracted. The lesion was then excised along the marked margins using monopolar electrocautery. The surgical specimen was submitted for intraoperative frozen‐section analysis, which confirmed tumor‐free margins. Hemostasis was subsequently achieved, and the reconstructive phase was initiated (Figure 3). The buccal mucosal defect was reconstructed using an island tongue flap, which provided successfully tension‐free covering of the defect while preserving the vascularized pedicle (Figure 4).

Subsequently, a 5 cm right submental transverse arcuate cervical incision was made, followed by the creation of a subplatysmal flap, and en bloc lymph node dissection involving right and left Levels IA at the neck level.

The surgical procedure was completed without complications. The patient was extubated with ventilatory autonomy, stable, and progressing clinically favorably. At the 15‐day postoperative follow‐up, a plan was established for lingual pedicle trimming and a committee evaluation for adjuvant treatment.

#### 2.4.2. Second Surgical Time

**Figure 5:**
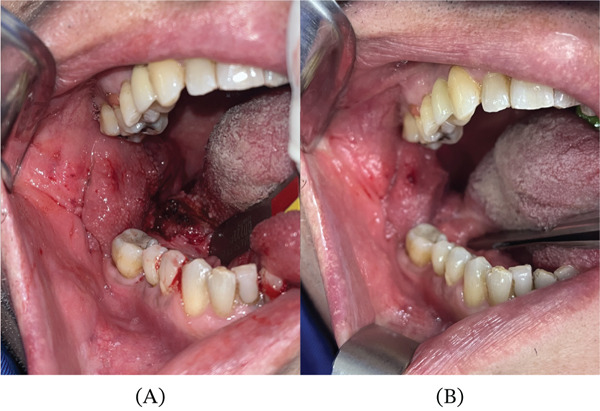
Reconstruction of oral defect. (A) Reconstruction of a right oral defect with a lingual island flap canalization. (B) Second surgical intervention: trimming of the lingual vascular pedicle.

In April 2025, the second surgical stage was carried out; the release of the tongue pedicle plus complex vestibuloplasty with muscle replacement was performed (Figure 5).

### 2.5. Committee Resolution

Due to local recurrence and considering the patient′s age, adjuvant radiotherapy to the tumor bed and ipsilateral cervical lymph node chains in combination with cetuximab was indicated.

During postoperative follow‐up, satisfactory intraoral healing was observed, with no evidence of flap necrosis, wound dehiscence, or scar contracture. The patient also demonstrated adequate tongue mobility, including protrusion, elevation, and lateralization, with satisfactory preservation of speech, swallowing function, and mouth opening.

**Figure 6:**
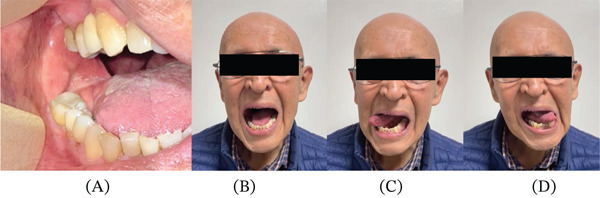
At the 14‐month clinical follow‐up. (A) Adequate intraoral healing and stable coverage of the reconstructed defect are observed. (B) Preserved mouth opening of 35 mm. (C) Tongue mobility during right lateralization. (D) Tongue mobility during left lateralization.

At the 14‐month follow‐up, the patient remained asymptomatic, with complete wound healing, adequate tissue coverage, and preserved tongue function. Clinical examination demonstrated good tongue mobility without significant functional impairment and no clinical evidence of local recurrence (Figure 6).

## 3. Discussion

Squamous cell carcinoma of the oral cavity is the most common malignant neoplasm in this region, accounting for approximately 90% of oral tumors. It originates from the squamous epithelium of the oral mucosa and is associated with risk factors such as tobacco use, alcohol consumption, and HPV infection [1, 2]. Its clinical presentation varies from leukoplakia or erythroplasty to indurated ulcers. The course is usually slow but progressive, with the potential for local invasion and regional metastasis. Early diagnosis is crucial for the prognosis, with incisional biopsy being the reference standard, whereas imaging techniques and complementary studies allow for the evaluation of the extent and planning of surgical treatment [3, 5].

In the present case, after surgical resection of the squamous cell carcinoma involving the buccal mucosa, posterior maxilla, palate, and retromolar trigone, the resulting defect was reconstructed using a lingual island flap. This technique allowed adequate intraoral coverage, preserving lingual function and ensuring reliable vascularization of the flap. Reconstruction with a lingual island flap was particularly useful due to the proximity of the surgical site and the limited extension of the defect, favoring rapid functional recovery and reducing the risk of postoperative complications [6].

Intraoral defects following oncologic resection with safety margins present complexities in reconstruction with similar tissue, using simpler techniques and decreasing the degree of morbidity. Intraoral flaps placed around the surgical bed offer adequate coverage depending on the size of the defect [4, 6]. Cho Wonseok et al. [6] reported that, compared with other intraoral flaps, the most notable advantages of the lingual flap lie in its technical simplicity and ease of execution, increased availability of effective reconstruction options for moderate‐sized defects, and preservation of lingual function.

Although microvascular free flaps currently represent the reconstructive gold standard for extensive oral cavity defects due to their versatility and ability to restore tissue volume, these procedures require longer operative times, specialized microsurgical expertise, and may be associated with greater donor‐site morbidity and prolonged hospital stays [7]. Similarly, the submental flap constitutes a reliable regional alternative for moderate‐sized defects; however, its use in oncologic patients remains controversial because of the potential risk of transferring metastatic disease from Level IA cervical lymph nodes and the possible proximity of the flap to tumor lymphatic drainage pathways [8, 9].

In the present case, the choice of an island tongue flap was based on the posterior location of the defect, the moderate size of the resection, and the need for adjacent tissue with similar characteristics, allowing adequate intraoral coverage with lower morbidity and functional preservation. Furthermore, this technique avoided the need for complex microsurgical procedures and reduced operative time. Although the requirement for a second surgery to divide the pedicle is a recognized disadvantage, several recent studies have demonstrated favorable functional outcomes in terms of speech, swallowing, and tongue mobility, particularly in carefully selected small‐ to moderate‐sized intraoral defects [6, 10].

Despite the limitations described for this type of reconstruction, several studies have reported favorable functional outcomes following the use of the island tongue flap [4]. Kumar et al. [11], in a retrospective study of 42 patients who underwent reconstruction with a tongue flap, reported that 71.42% achieved adequate mouth opening, good tongue mobility, and satisfactory speech outcomes. Although transient alterations in phonation, swallowing, tongue mobility, and scar formation related to pedicle division have been described, no significant functional impairment was observed in the present case, which demonstrated a favorable clinical course and adequate preservation of tongue function.

The clinical significance of this report lies in demonstrating that the island tongue flap remains a valid and functional reconstructive option for carefully selected posterior intraoral oncologic defects, particularly in settings where minimizing surgical morbidity and preserving oral function are important considerations.

## 4. Conclusions

OSCC continues to represent a locally aggressive malignancy that requires surgical resection with adequate oncologic margins and individualized reconstructive planning.

Although microvascular free flaps are widely used for the reconstruction of extensive defects, the island tongue flap may represent an effective alternative for selected small‐ to moderate‐sized posterior intraoral defects because of its reliable vascular supply, proximity to the recipient site, lower morbidity, and preservation of tongue function.

The present case demonstrates favorable clinical outcomes in terms of tissue coverage and oral function, highlighting the importance of selecting reconstructive techniques tailored to the characteristics of the defect and the patient′s individual conditions.

## Funding

No funding was received for this manuscript.

## Consent

Written informed consent was obtained from the patient for publication of this case report and any accompanying images.

## Conflicts of Interest

The authors declare no conflicts of interest.

## Data Availability

The data that support the findings of this study are available from the corresponding author upon reasonable request.

## References

[1] Hwang D. S. , Park J. , Kim U. K. , Park H. R. , Kim G. C. , and Ryu M. H. , Reconstruction of Cheek Mucosal Defect With a Buccal Fat Pad Flap in a Squamous Cell Carcinoma Patient: A Case Report and Literature Review, Maxillofacial Plastic and Reconstructive Surgery. (2018) 40, no. 1-5, 10.1186/s40902-018-0150-8, 29872648. PMC5968009 29872648

[2] González-Guevara M. B. , Linares-Vieyra C. , Castro-García M. E. , Muñiz-Lino M. A. , Abaroa-Chauvet C. , and Bello-Torrejón F. , Oral Squamous Cell Carcinoma. Case Report and Review of Literature, Revista medica del Instituto Mexicano del Seguro Social. (2022) 60, no. 1, 85–90, 35274916. 35274916 PMC10396044

[3] Abati S. , Bramati C. , Bondi S. , Lissoni A. , and Trimarchi M. , Oral Cancer and Precancer: A Narrative Review on the Relevance of Early Diagnosis, International Journal of Environmental Research and Public Health. (2020) 17, no. 24, 10.3390/ijerph17249160, 33302498. PMC7764090 33302498

[4] Scott-Wittenborn N. , D′Souza G. , Tewari S. , Rooper L. , Troy T. , Drake V. , Bigelow E. O. , Windon M. J. , Ryan W. R. , Ha P. K. , Kiess A. P. , Miles B. , Westra W. H. , Mydlarz W. K. , Eisele D. W. , and Fakhry C. , Prevalence of Human Papillomavirus in Head and Neck Cancers at Tertiary Care Centers in the United States Over Time, Cancer. (2022) 128, no. 9, 1767–1774, 10.1002/cncr.34124, 35132635. 35132635 PMC9007835

[5] Barsouk A. , Aluru J. S. , Rawla P. , Saginala K. , and Barsouk A. , Epidemiology, Risk Factors, and Prevention of Head and Neck Squamous Cell Carcinoma, Medical Sciences. (2023) 11, no. 2, 10.3390/medsci11020042, 37367741. PMC10304137 37367741

[6] Cho W. , Jang E. A. , and Kim K. N. , Single-Stage Peninsular-Shaped Lateral Tongue Flap for Personalized Reconstruction of Various Small-to Moderate-Sized Intraoral Defects: A Retrospective Case Series With Tongue Function Evaluation Using the Functional Intraoral Glasgow Scale, Journal of Personalized Medicine. (2023) 13, no. 12, 10.3390/jpm13121637. PMC10744366 38138864

[7] Tomar N. , Iyer M. , and Jain A. , An Analysis of Outcome and Complications of Microvascular Free Flap Head and Neck Reconstruction, International Journal of Medical and Oral Research. (2020) 3, no. 1, 1–3, 10.36106/gjra/8000123.

[8] Paydarfar J. A. , Kahng P. W. , Polacco M. A. , and Zhao W. , The Submental Island Flap in Head and Neck Reconstruction: A 10-Year Experience Examining Application, Oncologic Safety, and Role of Comorbidity, Laryngoscope Investigative Otolaryngology. (2022) 7, no. 2, 361–368, 10.1002/lio2.741, 35434339. 35434339 PMC9008180

[9] Khan Z. , Saleem M. , Khan I. , and Rahman M. F. , Functional Outcomes of Tongue Reconstruction After Cancer Extirpation in Different Flaps: Pedicled Versus Free Flaps: A Systematic Review, Plastic and Reconstructive Surgery – Global Open. (2025) 13, no. 10, e7100, 10.1097/GOX.0000000000007100. 41098423 PMC12520226

[10] Thompson J. A. , Vakharia K. T. , and Hatten K. M. , Advances in Oral Tongue Reconstruction: A Reconstructive Paradigm and Review of Functional Outcomes, Current Opinion in Otolaryngology & Head and Neck Surgery. (2022) 30, no. 5, 368–374, 10.1097/MOO.0000000000000828, 36004797. 36004797

[11] Kumar V. , Mukharjee S. , Akhtar N. , Rajan S. , Chaturvedi A. , Misra S. , Gupta S. , Prakash P. , and das S. , Tongue Flap Reconstruction in Carcinoma of Oral Cavity: An Institutional Experience, Journal of Maxillofacial and Oral Surgery. (2019) 18, no. 3, 428–431, 10.1007/s12663-018-1123-2, 31371886. 31371886 PMC6639513

